# Late diagnosis of anorectal malformation: how good is good enough?

**DOI:** 10.1007/s00383-024-05765-2

**Published:** 2024-07-05

**Authors:** Joseph Davidson, Indre Zaparackaite, Charlotte Holbrook, Hemanshoo Thakkar

**Affiliations:** 1 Department of Paediatric Surgery, Evelina London Children’s Hospital, London, SE1 7EH UK; 2 UCL Great Ormond Street Institute of Child Health, London, UK; 3 King’s College London School of Medicine, London, UK

**Keywords:** Anorectal malformation, Congenital anomaly, Newborn screening, Delayed diagnosis

## Abstract

**Purpose:**

National data from the United Kingdom reported in 2016 have suggested that almost one quarter of babies with anorectal malformation (ARM) have a delay in diagnosis. The UK’s Newborn Infant Physical Examination dictates a perineal examination should be performed within 72 h of birth. We sought to describe a tertiary single-centre experience of late presentation in the most recent 5 years.

**Methods:**

A single-centre prospective registry of ARM patients (July 2018–March 2024) was analysed. Timing of presentation with anomaly was noted. Patients presenting > 72 h or having been discharged home were defined as a delayed diagnosis. Factors associated with delayed diagnosis were noted.

**Results:**

Sixty patients were included, of whom nine (15%) were diagnosed after 72 h [range 4–279 days]. This represents a non-significant improvement compared to 39/174 (22%) late diagnosed cases in the BAPS-CASS cohort from 2016 to 17 (*p* = 0.188). Presenting symptoms of obstruction (i.e. distension, vomiting, megarectum) were more common in late diagnosed patients (4/9 (44%) vs. 1/51(2%); *p* = 0.001). Anomalies producing meconium on the perineum were more likely to be diagnosed late (8/32 (25%) vs 1/28 (4%); *p* = 0.029). Complications and changes to clinical management for these cases are presented.

**Conclusion:**

Although our regional rates of late diagnosis appear to be lower than previously reported national rates, there remains a significant number of infants who are diagnosed late especially those with visible perineal openings. These infants are more commonly symptomatic; entraining additional risks associated with an emergency presentation.

## Introduction

There is a wide variation of anatomy in anorectal malformations (ARMs) leading to patients not being immediately symptomatic or being identified during early life. Timely diagnosis is important, irrespective of the severity of the anomaly, to allow for optimal management with strategies to decompress the bowel and minimise the risk of complications resulting from obstruction [1–3]. There is undoubtedly also a psychological benefit to the timely diagnosis of patients, as incurable constipation may be a prominent symptom in a child who has not been correctly diagnosed in the newborn period.

Previous single-centre data have been compared between the United Kingdom and European Centres in the timely detection of ARMs in the newborn period and outlined that there may be a practice gap [4]. Furthermore, the proportion of cases that are missed was confirmed with the BAPS-CASS cohort data, recently published, suggesting that more than one in five (22%) infants with an ARM may be diagnosed late with 7% being detected after 1 month of age [5]. Since these datasets have been published, there has been a nationwide roll-out of changes to the Newborn and Infant Physical Examination (NIPE) which specifically directs the clinician to a thorough examination of the newborn perineum [6].

This study aimed to document the frequency of a delayed diagnosis of ARM, and explore potential associated patient factors with this. A secondary aim was to describe the clinical course for patients diagnosed beyond 72 h of age, outlining potential morbidity that may have been avoided.

## Methods

This was a single-centre study based upon a prospective registry of ARMs over a period of 4 years 8 months (August 2019–May 2024). Our centre receives referrals from a mixed urban/rural population of 5.5 million in the south-east of England.

Patient demographic data were noted as well as the place of birth (home/district hospital/specialist hospital). We noted whether patients had any associated anomalies and whether they had a VACTERL association (Vertebral, Anorectal, Cardiac, Tracheoesophageal, Renal and Limb).

### Definitions

We classified a delayed diagnosis as any patient who had been discharged from their centre of birth or was diagnosed after 72 h (beyond the time at which the NIPE is meant to have taken place).

VACTERL association was classified as having 2 anomalies additional to the ARM.

ARM type were classified as per the Wingspread classification. We have not included any cases of anterior ectopic anus (whereby an anus of normal calibre is situated entirely within a sphincter complex).

### Analysis

Each patient factor was explored for its association with a delayed diagnosis. Categorical data were compared by Chi-square of Fisher’s exact as appropriate for group sizing. We took a *p *value below 0.05 to denote statistical significance. Effect sizes for each are reported in the form of Relative Risk (RR) with accompanying 95% Confidence Interval (CI^95%^). Continuous data are presented as a median and range.

## Results

### Patient demographics and baseline characteristics

Over the study period, there were 60 new referrals to our centre with a diagnosis of ARM confirmed at examination under anaesthesia with electrical stimulation of the sphincter complex. Patient demographics and anomaly information are given in Table 1.

**Table 1:** Demographics and disease characteristics of included patients. Date presented as median [range] or n (%)

Total patients	60
Sex	
Male	41
Female	19
Age at presentation	0 [0–388] days
Type of anomaly (Male)	
Perineal fistula	12 (29%)
Rectourethral	13 (32%)
Rectoprostatic	5 (12%)
Rectobladder neck	2 (5%)
Anorectal atresia without fistula	5 (12%)
Anorectal stenosis	1 (2%)
Level not yet identified (not perineal)	3 (7%)
Type of anomaly (female)	
Perineal fistula	9 (47%)
Rectovestibular fistula	8 (42%)
Cloaca	2 (11%)
VACTERL (> 2 additional)	
Yes	9 (15%)
No	51 (85%)
Non-VACTERL major anomalies	
Yes	8 (13%)
No	52 (87%)

NB 3 male patients without a perineal opening have not yet had a distal contrast study to determine the level of their fistula

### Delayed diagnosis and associated factors

Of the 60 patients, 9 were diagnosed beyond 72 h at a median of 43 days [range 4–388]. Two presented within 1 week (4 and 7 days) with abdominal distension, the remaining seven patients all had perineal openings. Univariate analysis of factors that were associated with delayed diagnosis was performed using Fisher’s exact and an accompanying risk ratio was produced. Although female sex appeared to increase the risk of delayed diagnosis and being born within a surgical centre decreased the risk, neither of these factors met statistical significance. However, anomalies with meconium at the perineum (i.e. perineal fistula and vestibular fistula) were seven times more likely to have an associated delayed diagnosis (*p* = 0.029) (see Table 2).

**Table 2:** Univariate analysis of factors associated with a delayed diagnosis (> 72 h)

	Delayed diagnosis	RR [CI^95%^]	Fisher’s exact, *p*
Female vs male sex	5/19 vs 4/41	2.70 [0.81 – 8.93]	0.126
Meconium on perineum	8/32 vs 1/28	7.00 [0.93 – 52.56]	0.029
Surgical inborn	2/20 vs 7/40	0.57 [0.13 – 2.50]	0.704
VACTERL/major anomaly	3/16 vs 6/44	1.38 [0.39 – 4.86]	0.689

Taking the 15% observed delay in diagnosis amongst the patient cohort, this compared to a 22% (39/174) that was observed in the UK national cohort reporting from 2015 to 16 (*p* = 0.279). Recognising that the NIPE was introduced in 2023, we observed that, in fact, there were still 4/17 (24%) in the final 12 months of the study period (vs 5/43 prior to this (12%; *p* = 0.256).

In terms of the consequences of the delayed diagnosis, the initial operative management for each of the nine patients is described in Table 3. Whilst it cannot be said with certainty that all of the patients had an altered clinical course on account of their delayed diagnosis, there are certainly cases where this is the case:

- Patient 1 and Patient 3 both presented with signs of established lower GI obstruction and required emergency surgery out of hours to perform decompressive colostomy.
- Patient 5 presented with megarectum and required a colostomy at the time of their reconstruction. They suffered from a retraction of the lateral limb of their mucous fistula into the peritoneal cavity with washout fluid used during the surgery flowing retrograde and causing peritonitis, requiring an admission to the ICU with subsequent revision of the stoma (Clavien–Dindo IV-a Complication).
- Patient 8 had a missed anal stenosis that required a defunctioning ileostomy at 43 days of life for significant abdominal distension, high ileostomy output limited enteral feeding, resulting in a neonatal stay of more than 131 days.

**Table 3:** Patients in the cohort with a delayed diagnosis

Patient	Sex, age at presentation	Anomaly	Symptoms	Initial procedure	Stages of management
1	M, 4d	Rectourethral	LGI Obstruction	Colostomy	3-stage
2	F, 14d	Rectovestibular	Nil, observed by family	Colostomy	3-stage
3	M, 7d	Perineal	LGI Obstruction	Colostomy	3-stage
4	F, 18d	Rectovestibular	Nil, observed by health visitor	Colostomy	3-stage
5	F, 279d	Perineal	Constipation, megarectum	PSARP with Colostomy	2-stage^a^
6	M, 45d	Perineal	Constipation	Primary repair	1-stage
7	F, 171d	Perineal	Constipation	Primary repair	1-stage
8	M, 43d	Anorectal stenosis	Distension	Ileostomy	3-stage
9	F, 388d	Perineal	Constipation	Primary repair	1-stage

^a^Patient 5 had an early return to theatre due to abdominal sepsis from the formed colostomy

## Discussion

This study provides new data from a UK centre to demonstrate an ongoing issue with delayed diagnosis of ARM. Although our regional data show some improvement over national data from 5 years prior to the study period, there remains a significant number of patients who are diagnosed late and may be undergoing unnecessary procedures with associated morbidity.

Similar to previous studies, anomalies with a perineal opening are more likely to be missed [5], presumably because the presence of meconium in the nappy may be reassuring to care providers. Upon visual inspection, the anatomy may not be immediately obvious to the inexperienced clinician—who is often the team member tasked with routine checks of an otherwise well baby.

The implications of delayed diagnosis have been suggested previously and we have exhibited several such issues here—predominantly the need for defunctioning stoma in defects amenable to primary repair. Primary repair of perineal fistula outside of the neonatal period without a defunctioning stoma can present challenges as the passage of formed stools through the neo-anus increases the risk of wound infection and breakdown, which has prompted us to perform colostomy in several patients in this series [7].

The need for a stoma clearly necessitates at least one further abdominal procedure; however. the complications of neonatal stoma are known to exceed 25% [8]. There have also been documented mortalities following missed diagnosis of ARM as a result of intestinal perforation [9, 10].

This study benefits from prospectively collected data and presents contemporary results relevant to current screening guidelines as recommended by the health system in the United Kingdom. Its single-centre nature is a limitation, although the size of the catchment area and the number of centres referring across the region would suggest outcomes that are representative. Limited patient numbers may have limited the ability to perform sufficient analysis, increasing the likelihood of Type II statistical error.

Our centre sees a disproportionate number of VACTERL cases as it is also a supra-regional centre for foetal cardiology, and as such, there may be an over-representation of patients with associated anomalies. These patients may bias the results in both directions: on the one hand, patients requiring neonatal intensive care admission are more likely to be subjected to rigorous physical examination. Alternatively, the difficulties in diagnosing ARM antenatally, even with advanced foetal imaging methods such as MRI [11, 12], may mean that more subtle variants (such as anorectal stenosis or perineal fistula) may be overlooked whilst more obvious anomalies receive focus.

## Conclusion

These data provide a glimpse into modern-day clinical practice in the United Kingdom and concerningly continue to demonstrate a delay in diagnosis of ARM. We demonstrate that these children may need additional procedures that ought not be necessary for the anomaly with which they were born.

## Data Availability

No datasets were generated or analysed during the current study.
